# LPS promotes a monocyte phenotype permissive for human cytomegalovirus immediate-early gene expression upon infection but not reactivation from latency

**DOI:** 10.1038/s41598-017-00999-8

**Published:** 2017-04-11

**Authors:** V. G. Kew, M. R. Wills, M. B. Reeves

**Affiliations:** 1 Institute of Immunity & Transplantation, UCL Division of Infection & Immunity, Royal Free Hospital, London, NW3 2PF UK; 2 Department of Medicine, Addenbrooke’s Hospital, Cambridge, CB2 0QQ UK

## Abstract

Human cytomegalovirus (HCMV) infection of myeloid cells is closely linked with the differentiation status of the cell. Haematopoietic progenitors and CD14+ monocytes are usually non-permissive for lytic gene expression which can lead to the establishment of latent infections. In contrast, differentiation to macrophage or dendritic cell (DC) phenotypes promotes viral reactivation or renders them permissive for lytic infection. The observation that high doses of Lipopolysaccharide (LPS) drove rapid monocyte differentiation in mice led us to investigate the response of human monocytes to HCMV following LPS stimulation *in vitro*. Here we report that LPS triggers a monocyte phenotype permissiveness for lytic infection directly correlating with LPS concentration. In contrast, addition of LPS directly to latently infected monocytes was not sufficient to trigger viral reactivation which is likely linked with the failure of the monocytes to differentiate to a DC phenotype. Interestingly, we observe that this effect on lytic infection of monocytes is transient, appears to be dependent on COX-2 activation and does not result in a full productive infection. Thus LPS stimulated monocytes are partially permissive lytic gene expression but did not have long term impact on monocyte identity regarding their differentiation and susceptibility for the full lytic cycle of HCMV.

## Introduction

Primary infection with human cytomegalovirus is usually asymptomatic in the healthy host and results in the establishment of a lifelong latent infection. One major site of latency is the haematopoietic progenitor cell population resident in the bone marrow^1–6^. Although these cells give arise to multiple white blood cell populations the carriage of viral genomes is restricted to the myelo-monocytic lineage in healthy individuals^3, 7, 8^. Importantly, the differentiation of monocyte and dendritic cell (DC) precursors to a mature macrophage or DC phenotype is sufficient to induce lytic viral gene expression and, ultimately, result in the reactivation of infectious virus production^4, 9–12^. This differentiation-dependent control of HCMV latency and reactivation is also reflected in the permissiveness of these same cell types for lytic infection^13–17^ and thus understanding the contribution of cellular differentiation to HCMV infection in the myeloid lineage contributes to our understanding of the factors that control viral infection.

First identified by Steinman and colleagues in the 1970s, DCs are a minor population of professional antigen presenting cells (APCs) that are able to activate primary adaptive T cell immune response^18, 19^. A body of literature now reports the identification and characterisation of a number of sub-populations of DCs which can be differentiated from each other by cell surface markers, anatomical localisation and cytokine production as well as mechanistic differences which has largely been based on murine studies^20^. The paucity of circulating DCs in the peripheral blood compartment has led researchers to use *in vitro* differentiation of CD34+ or CD14+ precursors to study the function of these cells^21, 22^. These studies include HCMV where a number of laboratories have employed the *in vitro* differentiation of progenitor cells to document the pivotal role cellular differentiation plays in latency and reactivation as well as lytic infection^3, 4, 6, 11, 13, 17, 23–28^.

Studies of HCMV rely largely on the long term culture of experimentally infected CD34+ or CD14+ cells to study viral reactivation *in vitro*. Importantly, a number of these experimental observations have been re-capitulated *ex vivo* through analyses of naturally latent cell types. Specifically, CD34+ cells and monocytes are not sites of viral reactivation^1, 3, 4, 7^. In contrast, DCs isolated directly *ex vivo* from healthy seropositives display evidence of lytic IE transcription and, when co-cultured with HFFs, can support HCMV reactivation^12^. Thus the models utilising *in vitro* differentiation of myeloid precursors to DCs can be exploited to study HCMV reactivation. However, a caveat is that whilst *in vitro* systems are informative they rely on relatively long term culture of DC populations that are then, to some degree, mapped onto the ontogeny of DCs *in vivo*.

However, a provocative study in the murine model pointed towards the rapid activation of circulating monocytes to a DC phenotype following an endotoxin insult^29^. The underlying premise was to show that systemic delivery of high doses of the endotoxin lipopolysaccharide LPS generated a large expanded population of DCs within 2–3 days in these animals. This putative DC population was hypothesised to be derived from circulating monocytes consistent with monocytes retaining the capacity to differentiate into DCs *in vivo*. Given our own interest in the role of DCs in reactivation we thus asked whether human monocytes treated *in vitro* under the same conditions could trigger viral reactivation - potentially providing a rapid model for studying HCMV reactivation.

Here we report that treatment of monocytes with high concentrations of LPS prior to infection generated a cell type permissive for lytic immediate-early (IE) gene expression. The infection rate was LPS dose-dependent with higher doses resulting in increased numbers of cells being IE positive. However, unlike in DCs, the infection was abortive with little evidence of DNA replication or virus production evident in these cells. Furthermore, the LPS induced permissiveness for lytic infection was transient and was sensitive to COX-2 inhibition. In contrast, the stimulation of long term latently infected monocytes with LPS failed to trigger IE gene expression from latency. The basis for these differences could not be attributed to a global defect in the ability of latently infected monocyte populations to respond to LPS. These data support a hypothesis that multiple mechanisms unique to the regulation of latent (but not lytic) IE gene expression need to be overcome for reactivation to ensue in differentiated cell types.

## Results

### LPS promotes monocyte permissiveness for HCMV immediate early gene expression but not viral replication

**Figure 1:**
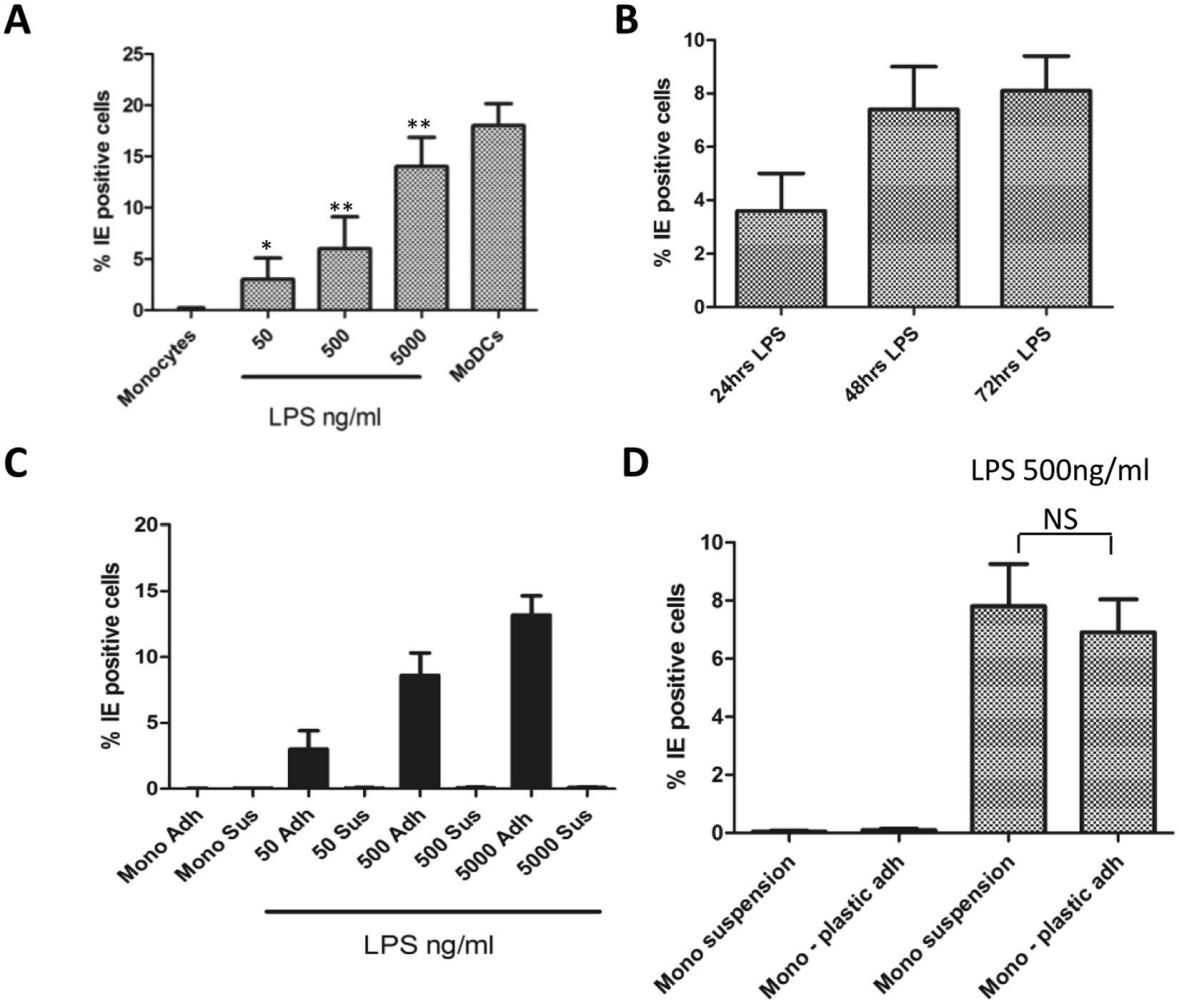
LPS promotes monocyte permissiveness in a dose dependent manner. (**A**) Monocytes were isolated from seronegative donors and incubated with mock, LPS (50–5000 ng/ml) or differentiated to DCs with IL-4/GMCSF. At 24 hours post LPS cells were infected with Merlin and then stained for IE protein expression 24 hpi. Nuclei were counter-stained with DAPI and infection rate calculated. Average of 3 donors shown. *p < 0.05, **p < 0.01; NS = non-significant difference when compared to infection of monocyte control. (**B**) Monocytes were incubated with 500 ng/ml of LPS. They were then infected at 24, 48 and 72 hours post LPS and then stained for IE protein expression 24 hpi. Nuclei were counter-stained with DAPI and infection rate calculated. Average of 3 donors shown. (**C**) Monocytes were incubated with 50–5000 ng/ml of LPS for 24 hours and then infected with Merlin. At 24hpi non-adherent cells were aspirated and cytospun onto slides. Both fractions were then stained for IE protein expression 24 hpi. Nuclei were counter-stained with DAPI and infection rate calculated. Average of 3 donors shown. (**D**) Monocytes were incubated either in non-adherent tubes or on plastic with media alone or 500 ng/ml of LPS for 24 hours and then infected with Merlin. At 24hpi the cell suspension was cytospun onto slides and all samples stained for IE protein expression 24 hpi. Nuclei were counter-stained with DAPI and infection rate calculated. Average of 3 donors shown. NS = non-significant difference.

CD14+ monocytes were isolated from healthy seronegative donors and stimulated with increasing concentrations of LPS. Three days post LPS stimulation, cells were infected with the Merlin strain of HCMV and analysed for IE protein expression by immuno-fluorescent microscopy 24 hours post infection. At the highest dose of LPS clear evidence of IE protein expression was observed in the monocytes (Fig. 1A). Log dilutions of LPS resulted in a correlative drop in HCMV infection suggesting that high doses of LPS triggered monocyte differentiation to a permissive phenotype. In these first studies two things became clear: the choice of HCMV strain had little impact since the same phenotype in these assays was seen with the Merlin and TB40/e strains and thus Merlin was used throughout and, secondly, addition of 5000 ng/ml of LPS resulted in a marked decrease in viability over time. Consequently, our studies focused on using 500 ng/ml of LPS where the phenotype was clear but the increased viability would not preclude more long term analyses of viral replication.

Having addressed the effect of dose we next addressed the impact of time on permissiveness. Thus cells were incubated with 500 ng/ml LPS and infected at 1–3 days post stimulation (Fig. 1B). Again we observed that the cells were permissive for HCMV infection and, furthermore, the longer the exposure to LPS prior to infection the greater their permissiveness.

Throughout these initial studies it was evident that a minor proportion of cells were loosely adherent and displayed fewer morphological changes associated with monocyte activation/differentiation (i.e. less granular and fewer dendritic-like processes). Thus we performed a second analysis where both the adherent and non-adherent fractions were analysed independently. Following infection, the loosely adherent cells were washed off and cytospun onto glass slides and stained for viral infection (Fig. 1C). As observed before, adherent LPS stimulated cells were permissive for HCMV infection whereas in contrast the non-adherent fraction were rarely IE positive (Fig. 1C) suggesting that adherence was possibly contributing to LPS-induced permissiveness. Thus to address whether adherence was directly important we repeated the analysis using non-adherent tubes. As before, monocytes were cultured with 500 ng/ml LPS for 24 hours and then infected with HCMV all in suspension. Cells were harvested, cytospun briefly onto glass slides and stained for IE protein expression 24hpi (Fig. 1D). In contrast, to the previous analysis where the minor (<1%) non-adherent fraction did not show evidence of lytic infection (Fig. 1C) a bulk analysis of LPS stimulated non-adherent monocytes showed the same dose dependent permissiveness observed with adherent monocyte cultures (Fig. 1A). Taken together, these data suggested that a minor fraction of the isolated monocyte population were inherently less permissive rather than adherence being an important determinant per se.

**Figure 2:**
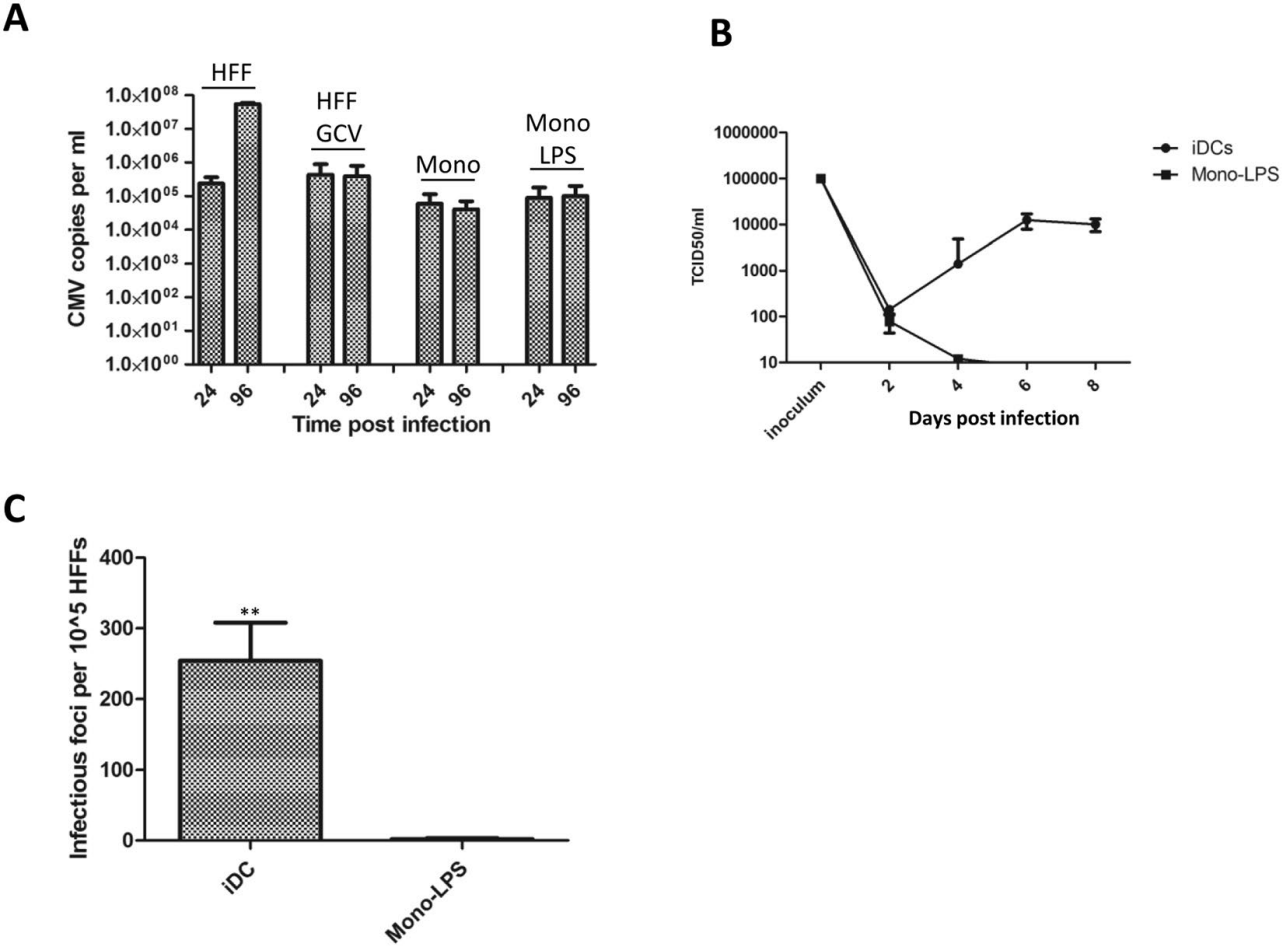
Infection of LPS stimulated monocytes is abortive. (**A**) Fibroblasts (HFFs), monocytes (mono) or LPS stimulated monocytes (Mono LPS) were infected with Merlin and then the DNA harvested at 24 and 96 hpi. Additionally, fibroblasts were incubated with ganciclovir (HFF GCV) at 24hpi. DNA was analysed in a diagnostic gB PCR to assay viral copy number n = 3. (**B**) Monocyte derived DCs (iDCs) or monocytes stimulated with 500 ng/ml LPS (Mono-LPS) were infected with Merlin (inoculum) and then samples of supernatants harvested every 2 days for analysis by TCID50 n = 2. (**C**) Monocyte derived DCs (iDC) or monocytes stimulated with 500 ng/ml LPS (Mono-LPS) were infected with Merlin (inoculum) and then after 6 days transferred to monolayers of HFFs which, after 2 further days, were stained for IE positive foci and enumerated per well. n = 2 **p < 0.01.

To address whether the observed expression of IE proteins was abortive or resulted in the production of infectious virus monocytes stimulated with LPS for 3 days were infected with HCMV and then analysed with a gB qPCR to measure viral DNA replication. HCMV DNA replicated in HFFs resulting in a 2.5 log increase in viral copy number from 24 to 96 hpi with replication being sensitive to ganciclovir (Fig. 2A). In contrast, no evidence for viral replication was observed in monocytes or monocytes pre-treated with LPS (Fig. 2A). A defect in DNA replication was consistent with a single step growth curve that demonstrated cell free infectious HCMV virions were not detected in the supernatant of infected LPS-mono cultures (Fig. 2B). Finally, to investigate the possibility that the LPS monos were producing only cell associated virus we co-cultured infected cells with HFFs at 6 days post infection for 2 days (Fig. 2C). Again the failure to detect infectious foci in the fibroblast monolayers provided further evidence that LPS stimulated monocytes were not capable of supporting the completion of the lytic HCMV lifecycle.

### High doses of LPS do not induce IE gene expression in latently infected monocytes

**Figure 3:**
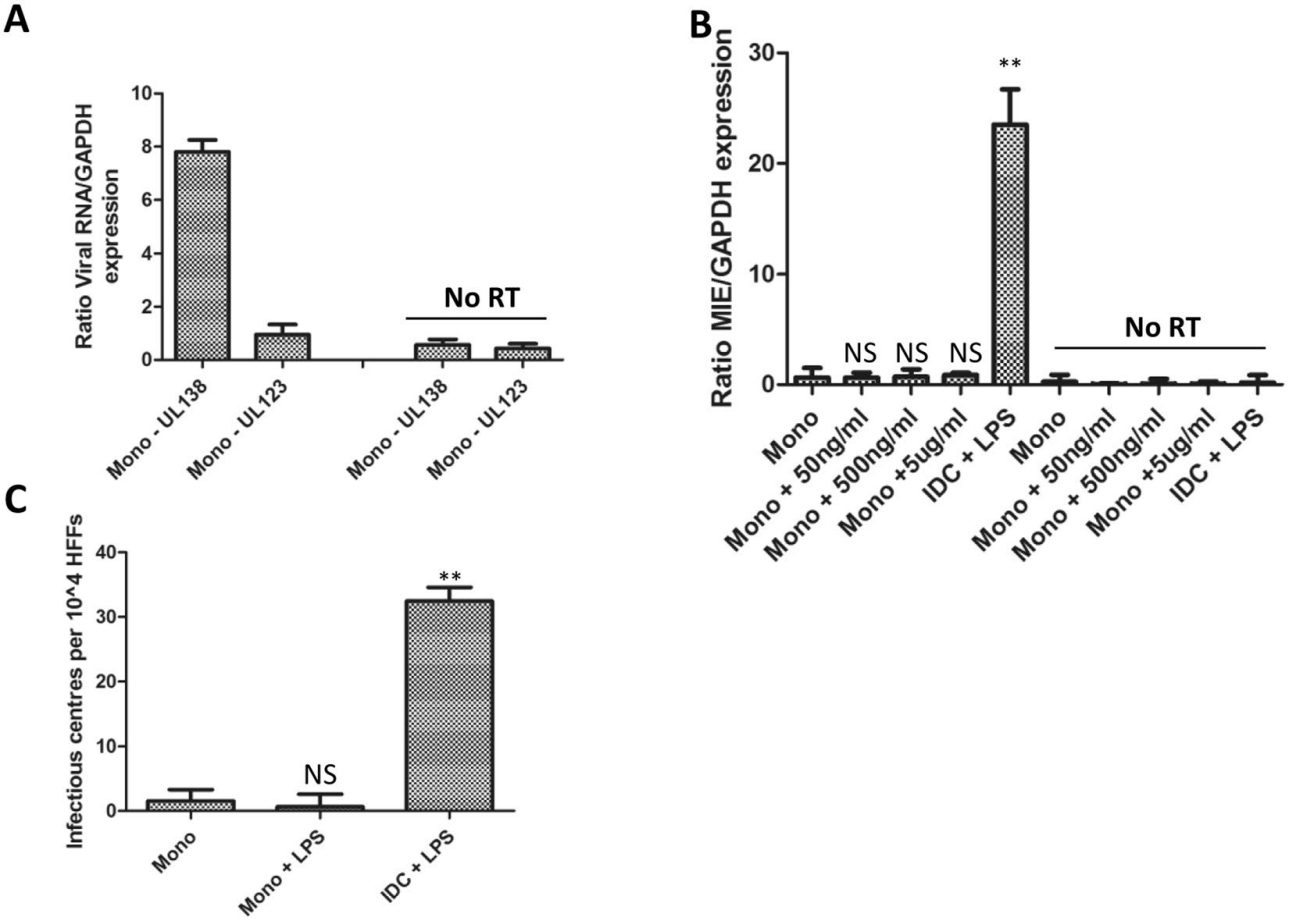
LPS does not induce IE gene expression in latently infected monocytes. **(A**) Monocytes were infected with Merlin and 3 dpi analysed for UL138 and UL123 expression by qRT-PCR and then calculated relative to GAPDH control. RNA without prior RT was analysed concurrently (no RT) n = 3. (**B**) Latently infected monocytes were either treated with media alone (mono), 50–5000 ng/ml LPS (Mono + LPS) or differentiated with IL-4/GM-CSF and then stimulated with 50 ng/ml LPS (iDC + LPS). Eight hours post stimulation, RNA was harvested and analysed for UL123 (MIE) gene expression and expressed relative to GAPDH. RNA without prior RT was analysed concurrently (no RT) n = 3. **p < 0.01; NS = non-significant difference when compared to monocyte control. (**C**) Latently infected monocytes were either treated with media alone (mono), 500 ng/ml LPS (Mono + LPS) or differentiated with IL-4/GM-CSF and then stimulated with 50 ng/ml LPS (iDC + LPS). Cells were then co-cultured with fibroblasts for 17 days which were stained for IE protein expression to identify infectious centres n = 3. **p < 0.01; NS = non-significant difference when compared to monocyte control.

A major research interest is to foster an understanding of the mechanisms that govern the induction of robust lytic IE gene expression from latently infected cells as the first stage required for HCMV reactivation. Thus we next tested whether induction of IE gene expression from latency was evident under culture conditions that drive monocyte permissiveness for lytic IE gene expression upon infection. Monocytes were infected with HCMV and were defined as latently infected by the concomitant detection of UL138 gene expression and minimal levels of major IE gene expression (Fig. 3A). These cells were then stimulated directly with 500 ng/ml LPS or, alternatively, with IL-4/GM-CSF to promote MoDC generation. We first analysed the induction of IE gene expression from latency (Fig. 3B). As expected, stimulation of immature MoDCs with LPS (50 ng/ml) was sufficient to trigger IE gene expression in these cells. In contrast, in monocytes not subject to prior differentiation no detectable IE gene expression was evident or when monocytes were incubated with LPS directly (Fig. 3B). This deficit in IE gene expression reflected in the level of HCMV reactivation where the co-culture of MoDCs but not LPS-monocytes resulted in the detection of infectious virus release into the cultures (Fig. 3C). Thus, although direct stimulation of monocytes with LPS was sufficient to promote permissiveness for lytic IE gene expression upon infection, it was insufficient to induce lytic IE gene expression and, consequently efficiently reactivate infectious virus, from long term latently infected monocytes.

### The secretome of latently infected monocytes does not antagonise LPS induced permissiveness in monocytes

The data thus far suggested that whilst LPS stimulation of monocytes rendered them permissive for lytic IE gene expression it was not sufficient for the induction of IE gene expression from latency. One possible explanation for these differences was that LPS induced effects on uninfected monocytes could occur through secondary messengers that are antagonised in latently infected cell cultures through modulation of the secretome^30, 31^.

**Figure 4:**
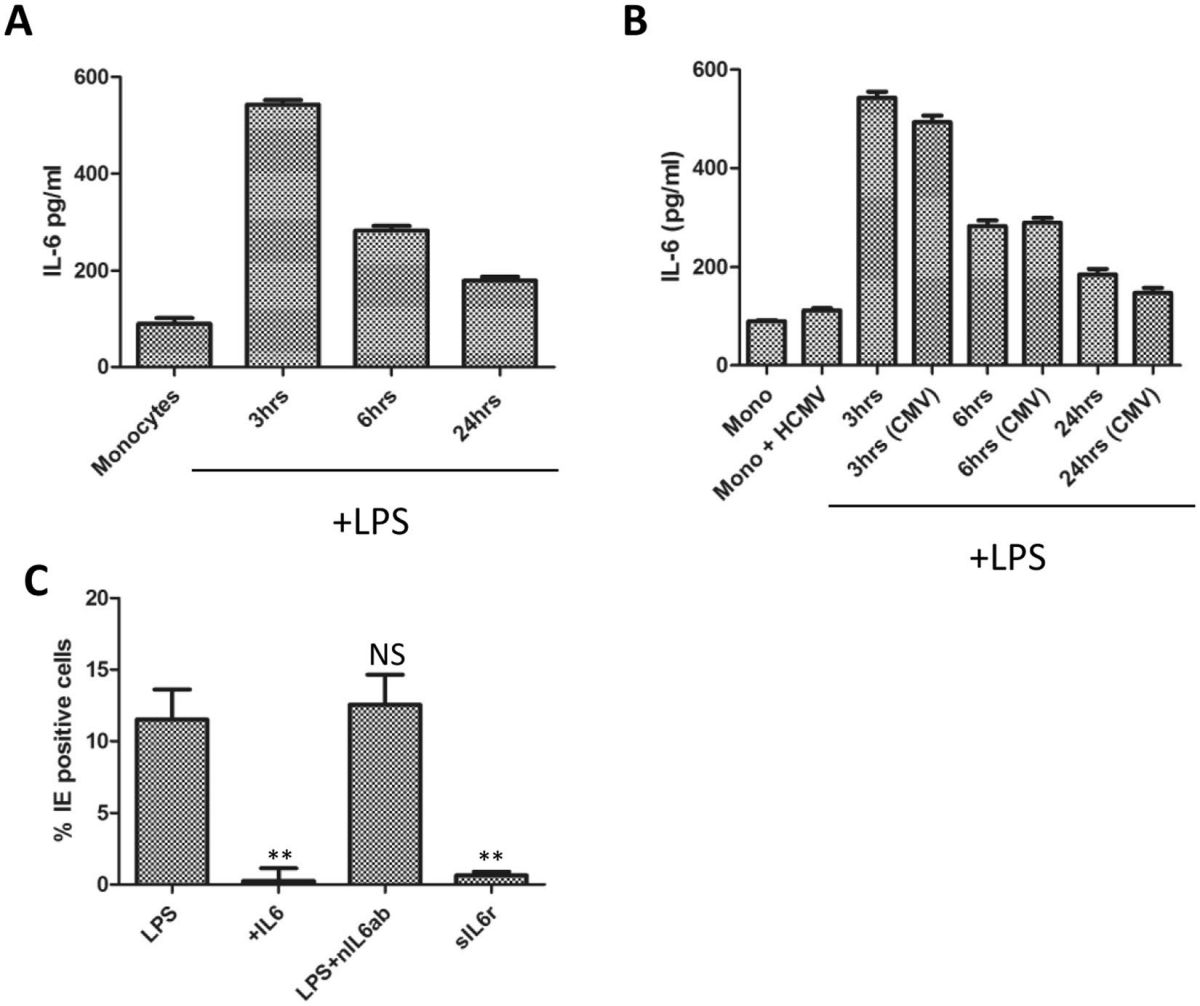
The differential effects of LPS are independent of IL-6 activity. (**A**) The supernatants from monocytes at 0, 3, 6 and 24 hours post LPS (500 ng/ml) stimulation were harvested and analysed for IL-6 production by ELISA n = 2. (**B**) The supernatants from uninfected monocytes (mono) or long term latently infected monocytes (Mono + HCMV) at 0, 3, 6 and 24 hours post LPS (500 ng/ml) stimulation were harvested and analysed for IL-6 production by ELISA n = 2. (**C**) Monocytes were stimulated with 500 ng/ml LPS, 50 ug/ml IL6, 500 ng/ml LPS plus nIL6 antibody, or 50 ug/ml soluble IL6 receptor (sIL6r) for 24 hours then infected with Merlin and then stained for IE protein expression 24 hpi. Nuclei were counter-stained with DAPI and infection rate calculated n = 3. **p < 0.01; NS = non-significant difference when compared to infection of LPS control.

We and others have shown that IL-6 – a cytokine induced by LPS – plays an important role in HCMV reactivation in DCs^32–34^ and long term monocyte cultures^35^ and, furthermore, IL-6 has been shown to play a role in the reprogramming of differentiation in monocytes^36^. Thus we asked whether differential IL-6 activity could explain the different outcomes observed during reactivation and primary infection. The first experiments addressed whether the lack of detectable reactivation in the LPS monocytes was simply linked with a decrease in IL-6 production in response to LPS stimulation and thus a reduction in paracrine signalling in the cultures. As expected, uninfected monocytes stimulated with LPS produced a burst of IL-6 - the levels of which decreased over time (Fig. 4A). An identical stimulation of latently infected monocyte cultures with LPS had no significant impact on the production of IL-6 in these cells over the same period of analysis (Fig. 4B) suggesting that an intrinsic failure in the capacity to generate IL-6 in the culture media could not explain a failure to reactivate in the cultures. Further support for many of these effects being IL-6 independent was provided by studies of permissiveness for lytic IE gene expression upon infection (Fig. 4C). Addition of either IL-6 or soluble IL-6 receptor to uninfected monocytes was not sufficient to pheno-copy the effect of LPS on monocyte susceptibility to lytic IE gene expression upon infection. Additionally, a neutralising antibody against IL-6 fails to reverse the ability of LPS to promote monocyte permissiveness for lytic IE gene expression (Fig. 4C). Taken together, the data provide no evidence that IL-6 plays an important role in generating the permissive phenotype nor are differences in IL-6 production the reason for a failure of LPS to trigger IE gene expression from latency in monocytes.

**Figure 5:**
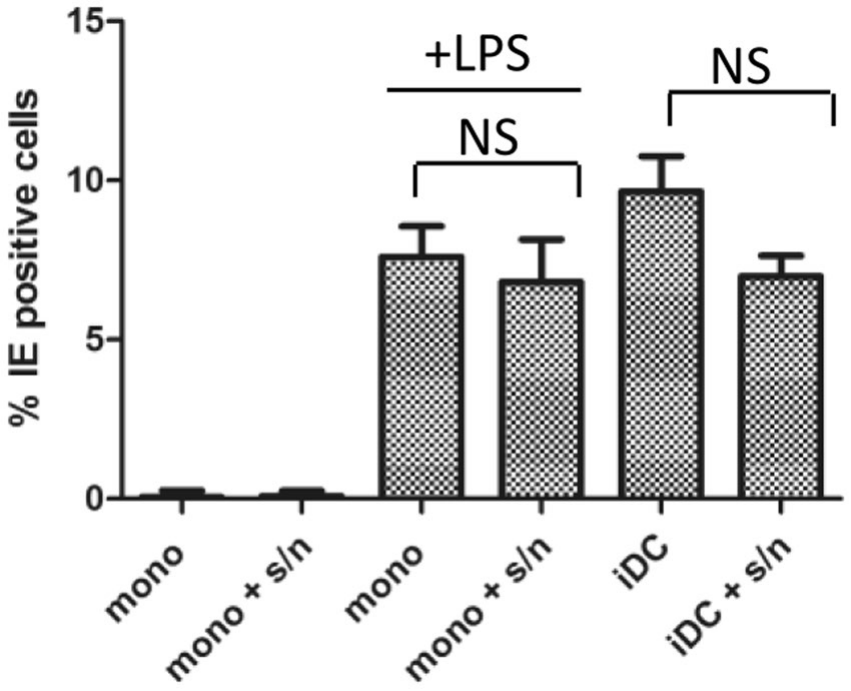
The supernatant of latently infected monocytes does not prevent LPS mediated effects on uninfected monocytes. To generate a latent secretome monocytes were infected with HCMV and cell free conditioned media harvested at 7dpi (s/n). Fresh monocytes were either incubated with normal (mono) or conditioned (mock + s/n) media for 48 hours. A fraction of the cells from both conditions were then further stimulated with (500 ng/ml LPS for 24 hours). Alternatively, uninfected monocytes were differentiated to permissive dendritic cells and incubated with normal media (iDC) or supernatant from latently infected monocytes (iDC + s/n) again for 48 hours. All cells were then infected with Merlin 2 days post incubation with mock or conditioned media and stained for IE protein expression 24 hpi. Nuclei were counter-stained with DAPI and infection rate calculated n = 3. NS = non-significant difference when comparing infection rates between mock and conditioned media treated cells.

Further experiments sought to address whether the secretome from latently infected cell cultures provided any potential cytokine mediated inhibition of LPS activity on monocytes. Supernatants from latently infected monocytes were harvested and incubated with fresh uninfected monocytes isolated from the same donors. After 48 hours monocyte cultures were stimulated with LPS and then subsequently tested their permissiveness for HCMV IE protein expression (Fig. 5). The data show that pre-incubation with the secretome of latently infected monocytes had little impact on the LPS phenotype: HCMV IE protein expression in these cells was comparable with control monocytes that had not been subject to prior incubation with the secretome from latently infected cells. Furthermore, consistent with the secretome of latently infected cells having an overt impact on the permissivity of myeloid cells for HCMV infection we demonstrated that the latent secretome did not prevent HCMV infection of monocyte derived DCs under the same experimental conditions. Thus the data could provide no evidence that the secretome from latently infected monocyte cultures contained a cytokine that blocked the ability of LPS to promote permissiveness for lytic IE gene expression in monocytes.

### LPS promotes a distinct phenotype inconsistent with myeloid differentiation

**Figure 6:**
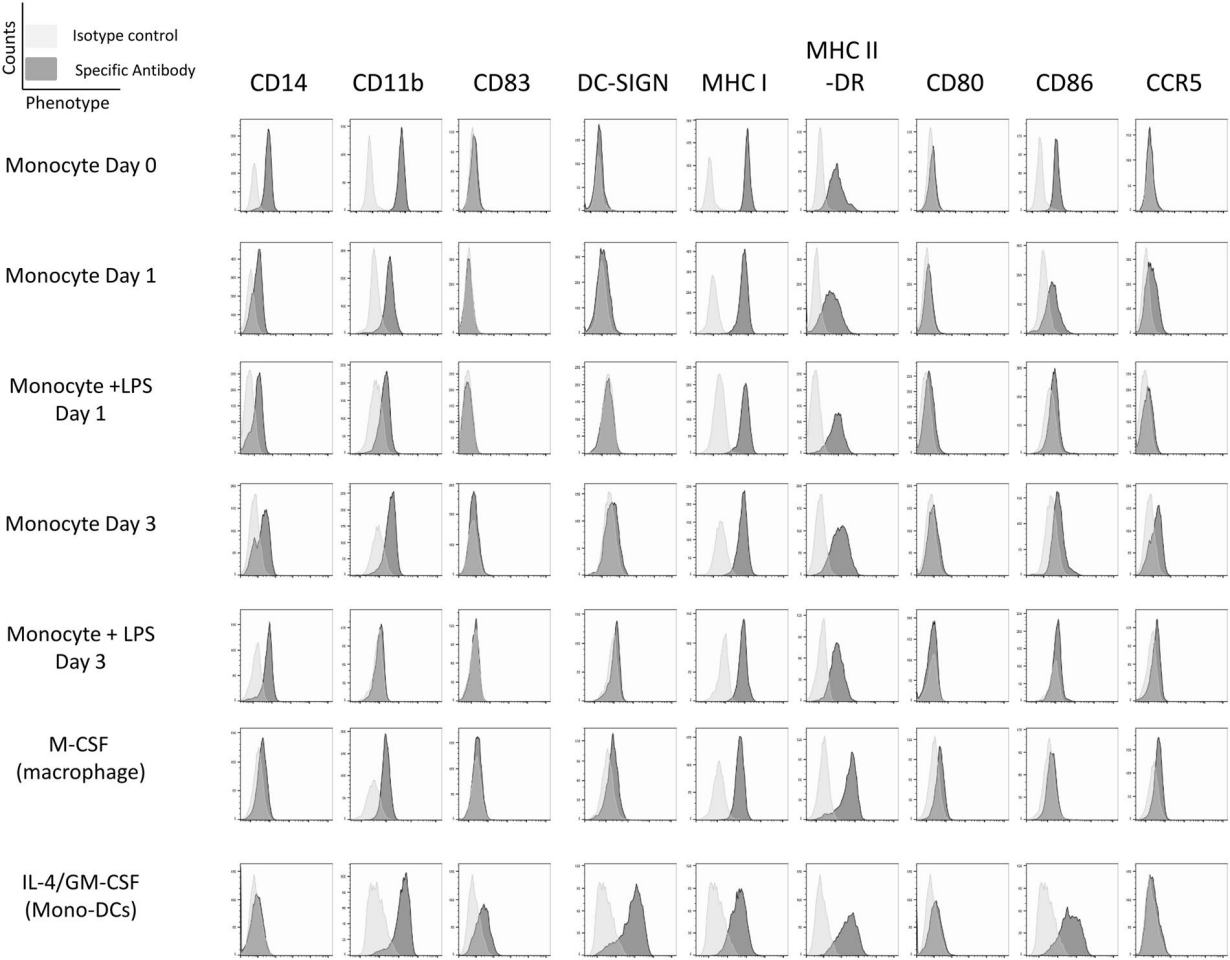
Incubation of monocytes with LPS promotes a unique phenotype. CD14+ monocytes were isolated by magnetic cell separation using CD14 beads from PBMC. Monocytes were incubated in media or stimulated with 500 ng/ml LPS for 1 and 3 days, IL4/GMCSF or MCSF for 6 days. Post incubation, monocytes were stained with cell surface antibodies to various phenotypic markers (CD14, CD83, CD86, CCR5, MHC Class II (HLA-DR), CD11b, CD209 (DC-SIGN), MHC Class I, CD80 or appropriate Isotype controls. Flow cytometry histogram plots against isotype controls for each treatment condition and phenotypic marker are shown.

We next considered the impact of LPS on the differentiation status of the monocytes. A comparative flow cytometric analysis of LPS-monocytes with unstimulated monocytes, classical MoDCs and M-CSF derived macrophages was performed (Fig. 6). The data clearly show that LPS does not induce a phenotype consistent with differentiation to a DC, evidenced by a lack of DC-SIGN expression or CD86 and HLA-DR upregulation, and the sustained expression of CD14. Interestingly, unlike M-CSF macrophages and DCs, addition of LPS directly to the monocytes promoted a dramatic loss of CD11b again suggesting a unique response. Furthermore, LPS monocytes expressed no detectable CD80, similar to monocytes, whereas the macrophages and DCs expressed, albeit modest, levels of CD80. Taken together, the phenotypic data provide no evidence to suggest that LPS was driving a more differentiated monocytes but, instead, bore more hallmarks of unstimulated monocytes in culture.

### The ability of LPS to generate a permissive environment for IE gene expression can be reversed

**Figure 7:**
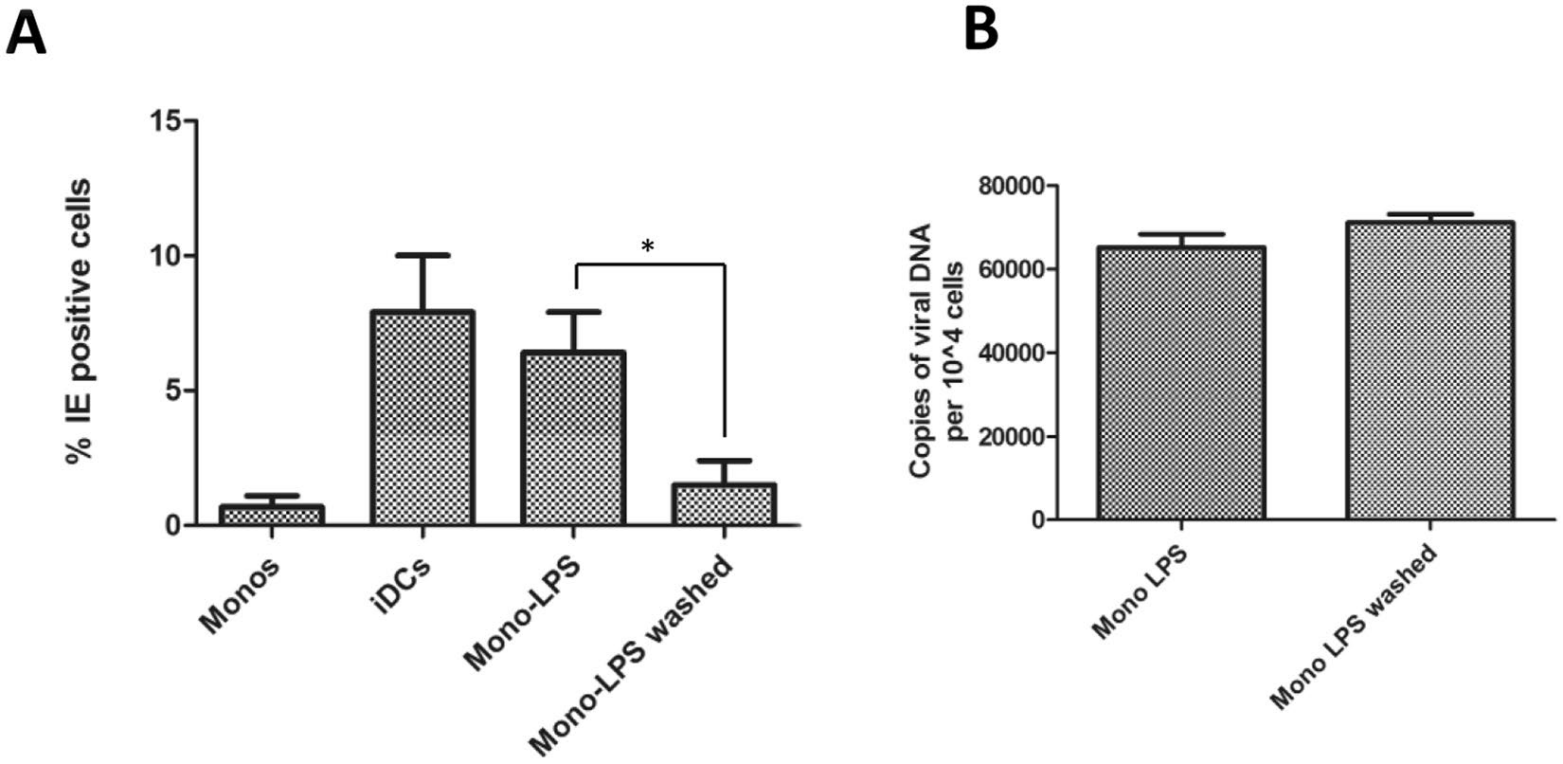
The LPS induced permissiveness of monocytes is transient. (**A**) Monocytes (monos) were either differentiated to dendritic cells (iDCs), stimulated with 500 ng/ml LPS for 72 hours (mono-LPS) or stimulated with LPS for 24 hours and then washed for 48 hours. Cells were then infected with Merlin and stained for IE protein expression 24 hpi. Nuclei were counter-stained with DAPI and infection rate calculated n = 3. (**B**) Monocytes were stimulated with 500 ng/ml LPS and then either incubated for 72 hours (Mono-LPS) or incubated for 24 hours followed by 48 hours in media alone (Mono LPS washed). DNA was then harvested and amplified in a diagnostic qPCR against gB with copy number normalised to cell number using a beta globin PCR n = 3. **p < 0.01 comparing infection in LPS and LPS-washed cells.

To further understand the differences we observed between lytic infection and HCMV reactivation in these analyses we next asked whether the LPS effect on monocyte susceptibility to lytic gene expression was permanent. The cell surface phenotyping suggested that LPS was not driving cellular differentiation based on the expression of a panel of cell surface markers that are known to be associated with classical DC differentiation. Also consistent with a lack of differentiation, was a failure to detect any evidence of viral reactivation from latency since, in a number of latency models, differentiation is a key determinant of reactivation. We hypothesised, therefore, that LPS could be promoting a priming event that is supportive of some lytic gene expression upon de novo infection. To test this, monocytes were incubated with 500 ng/ml LPS for 72 hours or, alternatively, incubated with LPS for 24 hours and then rescued in fresh media (48 hours) after multiple washes. At the 72 hour time point, the cells were then infected with HCMV and immuno-stained for IE gene expression (Fig. 7A). Unstimulated monocytes remained non-permissive for lytic IE gene expression whereas long term exposure to LPS permitted IE protein expression (Fig. 7A). Interestingly, short term exposure to LPS followed by a resuscitation period resulted in substantially fewer monocytes being permissive for lytic infection although some infection remained (Fig. 7A). This failure to initiate IE gene expression was not linked with an intrinsic block to viral entry. A PCR analysis of the cell DNA revealed similar levels of viral DNA in washed and unwashed LPS stimulated monocytes at 24 hours post infection (Fig. 7B).

**Figure 8:**
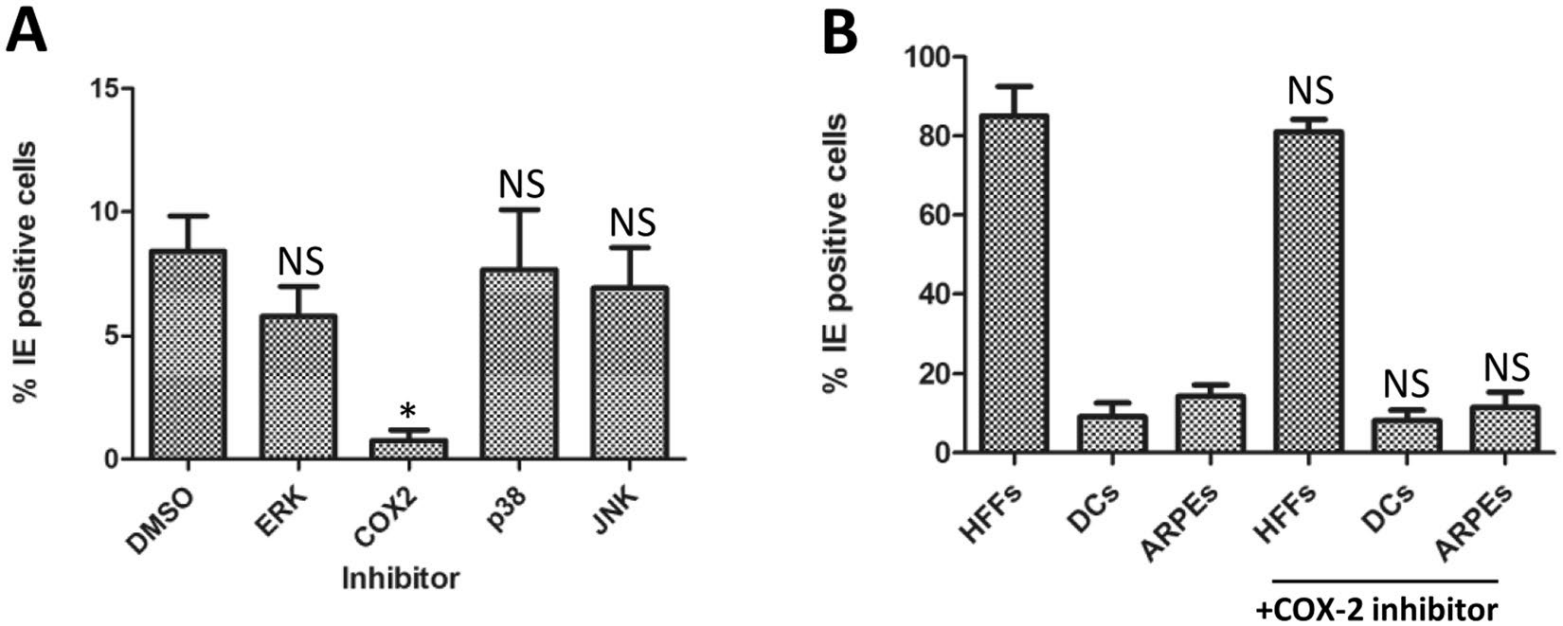
A COX-2 inhibitor antagonises LPS mediated permissiveness of monocytes. (**A**) Monocytes were incubated with control (DMSO), or inhibitors of ERK, COX2, p38 or JNK for 4 hours prior to addition of LPS (500 ng/ml). After 24 hours, cells were then infected with Merlin and stained for IE protein expression 24hpi. Nuclei were counter-stained with DAPI and infection rate calculated n = 3. **p < 0.01; NS = non-significant difference when compared to infection of DMSO solvent control. (**B**) HFFs, monocyte derived dendritic cells (DCs) or epithelial cells (ARPE) were pre-treated with DMSO or a COX-2 inhibitor for 4 hours then infected with Merlin and stained for IE protein expression 24hpi. Nuclei were counter-stained with DAPI and infection rate calculated n = 3. NS = non-significant difference when comparing rate of infection of each cell type plus and minus inhibitor.

A possible explanation for these transient effects was the hyper-activation of intracellular pathways that contribute to lytic infection during the initial stages of viral infection but are not capable of reversing viral latency. To investigate this further we tested the ability of a panel of inhibitors to block the LPS mediated effect on monocytes we had observed. Using inhibitors of downstream signalling pathways reported to be LPS responsive in various cell culture models we detected that the LPS induced phenotype was most potently inhibited by the COX-2 inhibitor, indomethacin (Fig. 8A). This COX-2 inhibitor phenotype was specific to LPS stimulated monocytes since pre-treatment of fully permissive cells with indomethacin had little impact on IE gene expression (Fig. 8B).

## Discussion

Permissiveness for lytic infection or for the reactivation of HCMV from latency in the myeloid lineage is intimately associated with the differentiation status of the cell^24^. In this study, we sought to address whether high doses of LPS could render normally non-permissive monocytes permissive for HCMV infection and reactivation. The rationale for this approach was driven by a previous study in the murine system which showed that circulating monocytes rapidly populated the DC pool in response to high concentrations of LPS^29^ – potentially representing a new, quicker approach to study HCMV reactivation *in vitro*.

The observation that high doses of LPS made normally non-permissive monocytes permissive for IE lytic gene expression led us to investigate further. However, the effects appeared to be transitory and much of the subsequent data argued against these cells being fully differentiated DCs *in vitro*. A caveat of initial analyses was the identification of a sub-population of ‘monocytes’ that appeared resistant to HCMV infection even if treated with LPS (Fig. 1). One possibility is that these are contaminating cell type and these cells were not analysed for CD14+ expression and no monocyte preparation will ever be 100% pure. However, an intriguing aspect was that an analysis by RT-PCR revealed that UL138 gene expression, but not UL123, could be detected. Thus this minor fraction could be infected but appeared to favour a latent infection phenotype.

Consistent with the infection data, phenotypic analyses suggested that although the specific changes in cell surface markers were evident the changes did not reflect a phenotype that could be described as a classical DC. Furthermore, the LPS stimulated monocytes, unlike classical DCs, did not support the production of new infectious particles arguing that an abortive infection was taking place. Finally, again consistent with an overt lack of differentiation, the addition of LPS to latently infected monocytes was not sufficient to promote reactivation. Related to the studies of HCMV reactivation we accept that in our supernatant experiments we cannot rule out the possibility that that latently infected cells are inherently less responsive to LPS since not all the cells within the population will be harbouring viral genomes. Thus although we could induce IL-6 in cultures of latently infected monocytes using LPS we cannot rule out that these effects are restricted to uninfected cells and that LPS (or IL-6) signalling is impaired in undifferentiated latently infected cells.

Central to our own research interests was the observation that the treatment of monocytes with LPS failed to trigger IE gene expression in latently infected cells. This clearly suggested that an intrinsic block to reactivation was not relieved under these differentiation/activation conditions in contrast to when the cells are incubated with IL-4/GM-CSF to make classical DCs^4, 32^. What this suggests is that the mechanisms governing the maintenance of long term latency are not wholly equivalent to the mechanisms important during the initial stages of infection in non-permissive cells. The mechanisms dictating long term latency are likely a concert of viral and cellular factors that promote the long term repression of lytic gene expression via the utilisation of histone modifying enzymes, chromatin structure, miRNA function and re-partitioning of cell signalling pathways^4, 37–43^. In contrast, during those initial stages the intrinsic hostile cellular environment the virus encounters – and likely a failure to overcome that - is the major determinant^44, 45^. Consequently, studying the basis of why the addition of LPS to latently infected monocytes does not drive viral reactivation can contribute to defining the mechanisms that do.

Why high doses of LPS makes monocytes permissive for IE gene expression is also an interesting question. It potentially could also explain studies which have suggested that monocytes and myeloid cells display a level of permissiveness for HCMV infection under certain conditions^46–48^. LPS is recognised by the pattern recognition receptor TLR4 which then signals downstream through multiple pathways^49^. HCMV has also been shown to bind and engage with TLRs – notably TLR2^50^ – but also TLR4 in monocytic cells^51^. Potentially, infecting monocytes at high MOIs may substitute for the LPS effects here and render monocytes transiently permissive for IE gene expression through delivery of high numbers of viral particles activating TLR4. It is possible that such a scenario could occur during viraemia and thus could represent a host response that is advantageous. The abortive expression of IE proteins in an absence of the expression of key immune evasion genes could facilitate a more effective T cell immune response and promote elimination of infected cells. It is this principle of inducing abortive IE protein expression that underpins the strategy of the use of HDAC inhibitors to clear latently infected cells^52^.

Alternatively, the addition of LPS could be impacting on the normal entry pathways active in the monocyte. Studies, mainly from the Yurochko laboratory, implicate the presence of a unique entry pathway into monocytes^53–56^. Most recently, they report that the HCMV genome can take upwards of 3 days to enter the nucleus^55^. Clearly, in these studies we are observing IE protein expression by 24 hours post infection and thus it is possible LPS is activating the cells to respond differently to infection circumventing this delayed pathway. Indeed, our observation that the LPS effect is COX-2 dependent in the monocytes may be linked to this. It has been previously described that COX-2 plays an important role in the replication and pathogenesis of CMV during lytic infection^57–59^. However, the major impact in fully permissive fibroblasts is on the switch from IE72 to IE86 protein expression and, ultimately, the production of new virions^58^. Thus during lytic replication COX-2 activation plays a key role during the later stages of viral infection and, importantly, post- IE and therefore cannot be directly related to the LPS effects we are reporting. However, a less well explored role of COX-2 is the potential of this pathway to impact on endocytosis and virus infection^60^. HCMV has evolved to utilise multiple entry pathways^61^ and thus the priming events we see with LPS may reflect a change in the pathway utilisation of HCMV due to hyper-activation of a COX-2 sensitive endocytic pathway in the monocytes. Although highly speculative, entry via this route may circumvent the usual entry pathway allowing the (potentially aberrant) initiation of viral gene expression. However, a post-IE block remains active in normally non-permissive cells and thus the virus does not continue into a full productive infection. An alternative, but linked, explanation may involve the delivery of tegument. A contributing factor to latent infection has been hypothesised to be the failure to deliver the major virion transactivator, pp71, to the nucleus in non-permissive myeloid cells^62^. Thus an equally plausible explanation could be that pp71 does traffic to the nucleus if the cells are primed with LPS.

Here we report the transient induction of a cellular environment in monocytes by LPS that supports IE lytic gene expression upon infection. In contrast, the effects of LPS on latent HCMV in monocytes is minimal suggesting that LPS fails to overcome additional blocks controlling IE gene expression during latency in monocytes. Understanding the molecular basis for these differences could enhance our understanding of the mechanisms governing HCMV reactivation. Importantly, we report that high doses of LPS *in vitro* do not appear to support the generation of a DC phenotype which is supported by both phenotypic evidence and, indirectly, the failure of HCMV to replicate in these cells. Therefore it is likely that LPS acts in concert with other cytokines *in vivo* to drive the DC phenotype observed in the murine model.

## Materials and Methods

### Ethical statement

All studies on monocytes were performed with local approval from either the Cambridge or UCL Local Research Ethics Committee and all experiments were performed under the guidelines and regulations set out by the committees. Informed consent was given locally unless excess material from NHS Blood Transfusion Service (Colindale, UK) was used where donors provide consent at point of donation for material to be used anonymously in research studies.

### Cells, tissue culture, cytokines and inhibitors

Human foreskin fibroblasts (HFFs) were maintained in Dulbecco’s minimal essential medium containing 10% fetal calf serum (DMEM-10) (GIBCO, UK) and incubated at 37 °C and in 5% CO_2_. CD14^+^ mononuclear cells were directly isolated from HCMV-seronegative blood and immature DCs were generated as described previously. Briefly, peripheral blood mononuclear cells (PBMCs) were prepared by centrifugation on a Ficoll-Hypaque (Lymphoprep; Takeda, UK) density gradient. Magnetic-activated cell sorting using CD14^+^ antibody-conjugated MicroBeads (Miltenyi Biotec, Surrey, UK) allowed collection of a CD14^+^ mononuclear cell-enriched population, which were maintained in X-VIVO 15 serum free media (Lonza, Walkersville, MD, USA). To promote differentiation to a MoDC phenotype, cultures were stimulated with IL-4 (100 ng/ml) and GM-CSF (100 ng/ml) for 6 days (both cytokines Peprotech, Rocky Hill, NJ). Loosely adherent cells were collected by moderate aspiration and transferred to fresh wells. Mature DCs were generated by adding LPS in fresh media (50 ng/ml; Sigma-Aldrich, Poole, UK).

Alternatively, monocytes were incubated directly with LPS (50–5000 ng/ml) for 1–3 days with no prior differentiation as described in the results. For experiments analyzing IL-6 activity, IL-6 (Peprotech, Rocky Hill, NJ; 500 ng/ml) and neutralizing IL6 antibody (R&D Systems, Minneapolis, MN; 10 ug/ml) were used at concentrations previously shown to have activity in HCMV reactivation^32^. The soluble IL6 receptor (Peprotech, Rocky Hill, NJ; 50 ng/ml) was used at an equivalent concentration to IL-6.

To measure IL-6 in the culture medium an IL-6 ELISA quantikine kit was used as described by manufacturer (R&D Systems, Minneapolis, MN).

The following inhibitors all Calbiochem (Merck, Darmstadt, Germany) unless stated were used at concentrations known to inhibit activity of their target. ERK (U0126;10 uM), JNK (SP600125; 5 uM), p38 (SB203580; 5 uM) and COX-2 (indomethacin; 1 uM; VWR international, Leicester, UK) were added to media 1 hour prior to stimulation with LPS and/or subsequent infection with HCMV. Ganciclovir (SIGMA, Poole, UK) was used at 10 ug/ml to inhibit viral DNA replication.

To culture TB40/e and Merlin strains of HCMV they were first cultured in ARPE-19 cells and then amplified following a single round of replication in HFFs. Virus was isolated and purified on sorbitol gradients as described previously^63^.

For DNA replication analysis experiments, infected monocytes were washed in PBS and then incubated for 1 hour with 2 mg/ml Proteinase K solution and the media replaced with fresh media to remove un-internalised virus from the cell surface.

### Latency establishment and co-culture experiments

To study latency CD14+ monocytes were cultured for at least 4 hours in X-vivo 15 following isolation and then infected with a preparation of the clinical isolate Merlin (MOI = 5 on fibroblasts; MOI = 0.1 in DCs). After 3 hours, infected cells are washed and cultured in fresh X-vivo 15 media for 7 days. After 3 days, media is exchanged again and fresh media containing cytokines that promote DC differentiation are added as described before. Virus production from reactivating mature MoDCs or from LPS stimulated monocytes was assayed by IE qRT-PCR 24 hours post stimulation or after following co-culture on a confluent monolayer of HFF with samples of supernatant taken at regular intervals and used to inoculate fresh HFF to test for infectious virus by indirect immuno-fluorescent staining.

### Indirect immunofluorescence and Western blotting

Infected cells were rinsed in PBS and fixed for 10 minutes in 4% paraformaldehyde at room temperature. After permeabilizing with 0.1% Triton-X-100 in PBS, cells were incubated with monoclonal mouse anti-IE antibody (Millipore) (1:1000 dilution in PBS) for 1 hour at room temperature. After washing with PBS, the bound antibodies were detected using Alexafluor 594 nm (Millipore, Billerica, MA)-conjugated goat anti-mouse immunoglobulins (1:1000 dilution in PBS) together with nuclear stain DAPI (300 nM in PBS) in the dark for 1 hour at room temperature. After washing with PBS, infected cells were visualised using a Nikon immunofluorescence microscope and were quantified using ImagePro WCIF ImageJ software (National Institutes of Health). Percentage infection was calculated by dividing the number of infected cells (red) by the total number of cells (blue) from at least 4 fields of view.

### Nucleic Acid Isolation and Analysis

Total RNA was extracted from 10^6^ cells using the RNAeasy kit as described by the manufacturer (Qiagen, Sussex, UK) Residual genomic DNA was removed by a DNAse I digestion (Promega, Madison, WI) followed by production of first-strand cDNA using the Promega RT system. For quantitative PCR, primers that amplified the IE region of HCMV were used^64^. The following reaction conditions were used in a 96 well plate format with forward primer AGC GCC GCA TTG AGG A, reverse primer CAG ACT CTC AGA GGA TCG GCC and probe ATC TGC ATG AAG GTC TTT GCC CAG TAC ATT (Fam probe with TAMRA quencher). PCR reactions were performed using Taqman MasterMix (Applied Biosystems, Foster City, CA) in a 7500HT machine (Applied Biosystems, Foster City, CA). Actin was amplified using a VIC-actin commercial probe (Applied Biosystems, Foster City, CA) and statistical analysis and interpretation performed as described^65^.

To assay DNA replication, total DNA was isolated using a sodium perchlorate method described previously which has been optimized for the isolation and detection of viral genomes from naturally latent mononuclear cells (23). Briefly, 10^6^ cells were resuspended in 600 ul of buffer A (100 mM NaCl, 5 mM; pH 8.0), lysed with 10% SDS (125 ul) and then incubated with 5 M sodium percholorate (150 ul). DNA was isolated by phenol:chloroform extraction and isopropanol precipitation. DNA copy number was then quantified using gB qPCR used in diagnostic assays^66^.

### Cell surface phenotype flow cytometry analysis

Monocyte at 10^6^ cells/ml were suspended in X-vivo 15 media in capped polypropylene FACS tubes (BD) pulsed with LPS, IL-4/GM-CSF to generate DCs or, 20 ng/ml M-CSF (Miltenyi Biotec, Surrey, UK) for 1, 3 or 6 days incubated at 37 °C 5% CO_2_. Cells were pelleted at 400 g for 5 minutes and 10^5^ cells per staining condition were stained with monoclonal antibodies specific for CD14-APC, CD83-APC, CD86-PE, CCR5-APC, MHC Class II (HLA-DR)-APC from BD Pharmingen (Oxford, UK), CD11b-FITC, CD209 (DC-SIGN)-PE, MHC Class I-PE from BioLegend (San Diego, CA) and CD80-APC from Abcam (Cambridge, UK). Isotype controls IgG1-FITC, IgG1-APC, IgG2a-PE, IgG2a-APC in the dark at room temperature for 30 mins.

Following washing in PBS, the cells were pelleted at 400 g for 5 minutes and were re-suspended in 500 μl of phosphate buffered saline (PBS) before analyzing by flow cytometry (BD AccuriC6). Data handling was performed using AccuriC6 and FLOWJO software.

### Statistical Analyses

Tests for statistical significance were performed using the non-parametric Mann Whitney U test on paired samples. Statistical significance was designated for any p value lower than 0.05.

## References

[1] Mendelson M, Monard S, Sissons P, Sinclair J (1996). Detection of endogenous human cytomegalovirus in CD34+ bone marrow progenitors. J Gen Virol.

[2] Sindre H, Tjoonnfjord GE, Rollag H, Ranneberg-Nilsen T, Veiby OP, Beck S, Degre M, Hestdal K (1996). Human cytomegalovirus suppression of and latency in early hematopoietic progenitor cells. Blood.

[3] Hahn G, Jores R, Mocarski ES (1998). Cytomegalovirus remains latent in a common precursor of dendritic and myeloid cells. Proc Natl Acad Sci USA.

[4] Reeves, M. B., MacAry, P. A., Lehner, P. J., Sissons, J. G. & Sinclair, J. H. Latency, chromatin remodeling, and reactivation of human cytomegalovirus in the dendritic cells of healthy carriers. *Proc Natl Acad Sci USA* **102**, 4140–4145, doi:040899410210.1073/pnas.0408994102 (2005). 10.1073/pnas.0408994102 PMC554799 15738399

[5] Dupont L, Reeves MB (2016). Cytomegalovirus latency and reactivation: recent insights into an age old problem. Rev Med Virol.

[6] Zhuravskaya T, Maciejewski JP, Netski DM, Bruening E, Mackintosh FR, St Jeor S (1997). Spread of human cytomegalovirus (HCMV) after infection of human hematopoietic progenitor cells: model of HCMV latency. Blood.

[7] Taylor-Wiedeman J, Sissons JG, Borysiewicz LK, Sinclair JH (1991). Monocytes are a major site of persistence of human cytomegalovirus in peripheral blood mononuclear cells. J Gen Virol.

[8] Bevan IS, Daw RA, Day PJ, Ala FA, Walker MR (1991). Polymerase chain reaction for detection of human cytomegalovirus infection in a blood donor population. Br J Haematol.

[9] Poole, E., Juss, J. K., Krishna, B., Herre, J., Chilvers, E. R. & Sinclair, J. Alveolar Macrophages Isolated Directly From Human Cytomegalovirus (HCMV)-Seropositive Individuals Are Sites of HCMV Reactivation *In Vivo*. *J Infect Dis* **211**, 1936–1942, doi:jiu83710.1093/infdis/jiu837 (2015). 10.1093/infdis/jiu837 PMC4442624 25552371

[10] Taylor-Wiedeman J, Sissons P, Sinclair J (1994). Induction of endogenous human cytomegalovirus gene expression after differentiation of monocytes from healthy carriers. J Virol.

[11] Soderberg-Naucler, C., Fish, K. N. & Nelson, J. A. Reactivation of latent human cytomegalovirus by allogeneic stimulation of blood cells from healthy donors. *Cell* **91**, 119–126, doi:S0092-8674(01)80014-3 (1997). 10.1016/s0092-8674(01)80014-3 9335340

[12] Reeves, M. B. & Sinclair, J. H. Circulating dendritic cells isolated from healthy seropositive donors are sites of human cytomegalovirus reactivation *in vivo*. *J Virol* **87**, 10660–10667, doi:JVI.01539-1310.1128/JVI.01539-13 (2013). 10.1128/JVI.01539-13 PMC3807413 23885077

[13] Riegler S, Hebart H, Einsele H, Brossart P, Jahn G, Sinzger C (2000). Monocyte-derived dendritic cells are permissive to the complete replicative cycle of human cytomegalovirus. J Gen Virol.

[14] Lathey JL, Spector SA (1991). Unrestricted replication of human cytomegalovirus in hydrocortisone-treated macrophages. J Virol.

[15] Gonczol E, Andrews PW, Plotkin SA (1984). Cytomegalovirus replicates in differentiated but not in undifferentiated human embryonal carcinoma cells. Science.

[16] Weinshenker BG, Wilton S, Rice GP (1988). Phorbol ester-induced differentiation permits productive human cytomegalovirus infection in a monocytic cell line. J Immunol.

[17] Hertel L, Lacaille VG, Strobl H, Mellins ED, Mocarski ES (2003). Susceptibility of immature and mature Langerhans cell-type dendritic cells to infection and immunomodulation by human cytomegalovirus. J Virol.

[18] Steinman RM, Cohn ZA (1973). Identification of a novel cell type in peripheral lymphoid organs of mice. I. Morphology, quantitation, tissue distribution. J Exp Med.

[19] Steinman RM, Lustig DS, Cohn ZA (1974). Identification of a novel cell type in peripheral lymphoid organs of mice. 3. Functional properties in vivo. J Exp Med.

[20] Schraml, B. U. & Reis e Sousa, C. Defining dendritic cells. *Curr Opin Immunol* **32**, 13–20, doi:S0952-7915(14)00148-410.1016/j.coi.2014.11.001 (2015). 10.1016/j.coi.2014.11.001 25553392

[21] Strobl H, Bello-Fernandez C, Riedl E, Pickl WF, Majdic O, Lyman SD, Knapp W (1997). flt3 ligand in cooperation with transforming growth factor-beta1 potentiates *in vitro* development of Langerhans-type dendritic cells and allows single-cell dendritic cell cluster formation under serum-free conditions. Blood.

[22] Sallusto F, Lanzavecchia A (1994). Efficient presentation of soluble antigen by cultured human dendritic cells is maintained by granulocyte/macrophage colony-stimulating factor plus interleukin 4 and downregulated by tumor necrosis factor alpha. J Exp Med.

[23] Reeves, M. B., Lehner, P. J., Sissons, J. G. & Sinclair, J. H. An *in vitro* model for the regulation of human cytomegalovirus latency and reactivation in dendritic cells by chromatin remodelling. *J Gen Virol* **86**, 2949–2954, doi:86/11/294910.1099/vir.0.81161-0 (2005). 10.1099/vir.0.81161-0 16227215

[24] Sinclair J, Reeves M (2014). The intimate relationship between human cytomegalovirus and the dendritic cell lineage. Front Microbiol.

[25] Lee, A. W., Hertel, L., Louie, R. K., Burster, T., Lacaille, V., Pashine, A., Abate, D. A., Mocarski, E. S. & Mellins, E. D. Human cytomegalovirus alters localization of MHC class II and dendrite morphology in mature Langerhans cells. *J Immunol* **177**, 3960–3971, doi:177/6/3960 (2006). 10.4049/jimmunol.177.6.3960 16951359

[26] Lauron, E. J., Yu, D., Fehr, A. R. & Hertel, L. Human cytomegalovirus infection of langerhans-type dendritic cells does not require the presence of the gH/gL/UL128-131A complex and is blocked after nuclear deposition of viral genomes in immature cells. *J Virol* **88**, 403-416, doi:JVI.03062-1310.1128/JVI.03062-13 (2013). 10.1128/JVI.03062-13 PMC3911714 24155395

[27] Moutaftsi M, Mehl AM, Borysiewicz LK, Tabi Z (2002). Human cytomegalovirus inhibits maturation and impairs function of monocyte-derived dendritic cells. Blood.

[28] Ibanez CE, Schrier R, Ghazal P, Wiley C, Nelson JA (1991). Human cytomegalovirus productively infects primary differentiated macrophages. J Virol.

[29] Cheong, C. *et al*. Microbial stimulation fully differentiates monocytes to DC-SIGN/CD209(+) dendritic cells for immune T cell areas. *Cell* **143**, 416–429, doi:S0092-8674(10)01125-610.1016/j.cell.2010.09.039 (2010). 10.1016/j.cell.2010.09.039 PMC3150728 21029863

[30] Avdic, S., Cao, J. Z., Cheung, A. K., Abendroth, A. & Slobedman, B. Viral interleukin-10 expressed by human cytomegalovirus during the latent phase of infection modulates latently infected myeloid cell differentiation. *J Virol* **85**, 7465–7471, doi:JVI.00088-1110.1128/JVI.00088-11 (2011). 10.1128/JVI.00088-11 PMC3126599 21593144

[31] Mason, G. M., Poole, E., Sissons, J. G., Wills, M. R. & Sinclair, J. H. Human cytomegalovirus latency alters the cellular secretome, inducing cluster of differentiation (CD)4+ T-cell migration and suppression of effector function. *Proc Natl Acad Sci USA* **109**, 14538–14543, doi:120483610910.1073/pnas.1204836109 (2012). 10.1073/pnas.1204836109 PMC3437838 22826250

[32] Reeves, M. B. & Compton, T. Inhibition of inflammatory interleukin-6 activity via extracellular signal-regulated kinase-mitogen-activated protein kinase signaling antagonizes human cytomegalovirus reactivation from dendritic cells. *J Virol* **85**, 12750–12758, doi:JVI.05878-1110.1128/JVI.05878-11 (2011). 10.1128/JVI.05878-11 PMC3209367 21937636

[33] Huang, M. M., Kew, V. G., Jestice, K., Wills, M. R. & Reeves, M. B. Efficient human cytomegalovirus reactivation is maturation dependent in the Langerhans dendritic cell lineage and can be studied using a CD14+ experimental latency model. *J Virol* **86**, 8507–8515, doi:JVI.00598-1210.1128/JVI.00598-12 (2012). 10.1128/JVI.00598-12 PMC3421708 22647696

[34] Carlier, J. *et al*. Paracrine inhibition of GM-CSF signaling by human cytomegalovirus in monocytes differentiating to dendritic cells. *Blood* **118**, 6783–6792, doi:blood-2011-02-33795610.1182/blood-2011-02-337956 (2012). 10.1182/blood-2011-02-337956 22031867

[35] Hargett, D. & Shenk, T. E. Experimental human cytomegalovirus latency in CD14+ monocytes. *Proc Natl Acad Sci USA* **107**, 20039–20044, doi:101450910710.1073/pnas.1014509107 (2010). 10.1073/pnas.1014509107 PMC2993366 21041645

[36] Chomarat P, Banchereau J, Davoust J, Palucka AK (2000). IL-6 switches the differentiation of monocytes from dendritic cells to macrophages. Nat Immunol.

[37] Rossetto, C. C., Tarrant-Elorza, M. & Pari, G. S. Cis and trans acting factors involved in human cytomegalovirus experimental and natural latent infection of CD14 (+) monocytes and CD34 (+) cells. *PLoS Pathog* **9**, e1003366, doi:10.1371/journal.ppat.1003366 PPATHOGENS-D-12-02509 (2013). 10.1371/journal.ppat.1003366 PMC3662700 23717203

[38] Buehler, J. *et al*. Opposing Regulation of the EGF Receptor: A Molecular Switch Controlling Cytomegalovirus Latency and Replication. *PLoS Pathog* **12**, e1005655, doi:10.1371/journal.ppat.1005655 PPATHOGENS-D-15-02781 (2016). 10.1371/journal.ppat.1005655 PMC4878804 27218650

[39] Lee SH, Albright ER, Lee JH, Jacobs D, Kalejta RF (2015). Cellular defense against latent colonization foiled by human cytomegalovirus UL138 protein. Sci Adv.

[40] Keyes, L. R., Hargett, D., Soland, M., Bego, M. G., Rossetto, C. C., Almeida-Porada, G. & St Jeor, S. HCMV protein LUNA is required for viral reactivation from latently infected primary CD14(+) cells. *PLoS One* **7**, e52827, doi:10.1371/journal.pone.0052827 PONE-D-11-22739 (2013). 10.1371/journal.pone.0052827 PMC3530514 23300789

[41] Murphy, E., Vanicek, J., Robins, H., Shenk, T. & Levine, A. J. Suppression of immediate-early viral gene expression by herpesvirus-coded microRNAs: implications for latency. *Proc Natl Acad Sci USA* **105**, 5453–5458, doi:071191010510.1073/pnas.0711910105 (2008). 10.1073/pnas.0711910105 PMC2291141 18378902

[42] O’Connor, C. M., Vanicek, J. & Murphy, E. A. Host microRNA regulation of human cytomegalovirus immediate early protein translation promotes viral latency. *J Virol* **88**, 5524–5532, doi:JVI.00481-1410.1128/JVI.00481-14 (2014). 10.1128/JVI.00481-14 PMC4019081 24599990

[43] Kew, V. G., Yuan, J., Meier, J. & Reeves, M. B. Mitogen and stress activated kinases act co-operatively with CREB during the induction of human cytomegalovirus immediate-early gene expression from latency. *PLoS Pathog* **10**, e1004195, doi:10.1371/journal.ppat.1004195 PPATHOGENS-D-13-02180 (2014). 10.1371/journal.ppat.1004195 PMC4055774 24945302

[44] Kalejta RF (2008). Functions of human cytomegalovirus tegument proteins prior to immediate early gene expression. Curr Top Microbiol Immunol.

[45] Saffert, R. T. & Kalejta, R. F. Inactivating a cellular intrinsic immune defense mediated by Daxx is the mechanism through which the human cytomegalovirus pp71 protein stimulates viral immediate-early gene expression. *J Virol* **80**, 3863–3871, doi:80/8/386310.1128/JVI.80.8.3863-3871.2006 (2006). 10.1128/JVI.80.8.3863-3871.2006 PMC1440479 16571803

[46] Rice GP, Schrier RD, Oldstone MB (1984). Cytomegalovirus infects human lymphocytes and monocytes: virus expression is restricted to immediate-early gene products. Proc Natl Acad Sci USA.

[47] Frascaroli, G., Varani, S., Moepps, B., Sinzger, C., Landini, M. P. & Mertens, T. Human cytomegalovirus subverts the functions of monocytes, impairing chemokine-mediated migration and leukocyte recruitment. *J Virol* **80**, 7578–7589, doi:80/15/757810.1128/JVI.02421-05 (2006). 10.1128/JVI.02421-05 PMC1563711 16840337

[48] Larsson S, Soderberg-Naucler C, Moller E (1998). Productive cytomegalovirus (CMV) infection exclusively in CD13-positive peripheral blood mononuclear cells from CMV-infected individuals: implications for prevention of CMV transmission. Transplantation.

[49] Akira S, Takeda K (2004). Toll-like receptor signalling. Nat Rev Immunol.

[50] Boehme, K. W., Guerrero, M. & Compton, T. Human cytomegalovirus envelope glycoproteins B and H are necessary for TLR2 activation in permissive cells. *J Immunol* **177**, 7094–7102, doi:177/10/7094 (2006). 10.4049/jimmunol.177.10.7094 17082626

[51] Yew, K. H., Carpenter, C., Duncan, R. S. & Harrison, C. J. Human cytomegalovirus induces TLR4 signaling components in monocytes altering TIRAP, TRAM and downstream interferon-beta and TNF-alpha expression. *PLoS One* **7**, e44500, doi:10.1371/journal.pone.0044500 PONE-D-12-01461 (2012). 10.1371/journal.pone.0044500 PMC3436894 22970235

[52] Krishna, B. A., Lau, B., Jackson, S. E., Wills, M. R., Sinclair, J. H. & Poole, E. Transient activation of human cytomegalovirus lytic gene expression during latency allows cytotoxic T cell killing of latently infected cells. *Sci Rep* **6**, 24674, doi:srep2467410.1038/srep24674 (2016). 10.1038/srep24674 PMC4835774 27091512

[53] Nogalski, M. T., Chan, G., Stevenson, E. V., Gray, S. & Yurochko, A. D. Human cytomegalovirus-regulated paxillin in monocytes links cellular pathogenic motility to the process of viral entry. *J Virol* **85**, 1360–1369, doi:JVI.02090-1010.1128/JVI.02090-10 (2010). 10.1128/JVI.02090-10 PMC3020497 21084488

[54] Nogalski, M. T., Chan, G. C., Stevenson, E. V., Collins-McMillen, D. K. & Yurochko, A. D. The HCMV gH/gL/UL128-131 complex triggers the specific cellular activation required for efficient viral internalization into target monocytes. *PLoS Pathog* **9**, e1003463, doi:10.1371/journal.ppat.1003463 PPATHOGENS-D-12-00173 (2013). 10.1371/journal.ppat.1003463 PMC3708883 23853586

[55] Kim, J. H., Collins-McMillen, D., Caposio, P. & Yurochko, A. D. Viral binding-induced signaling drives a unique and extended intracellular trafficking pattern during infection of primary monocytes. *Proc Natl Acad Sci USA* **113**, 8819–8824, doi:160431711310.1073/pnas.1604317113 (2016). 10.1073/pnas.1604317113 PMC4978277 27432979

[56] Chan, G., Nogalski, M. T. & Yurochko, A. D. Activation of EGFR on monocytes is required for human cytomegalovirus entry and mediates cellular motility. *Proc Natl Acad Sci USA* **106**, 22369–22374, doi:090878710610.1073/pnas.0908787106 (2009). 10.1073/pnas.0908787106 PMC2799688 20018733

[57] Hooks, J. J., Chin, M. S., Srinivasan, K., Momma, Y., Hooper, L. C., Nagineni, C. N., Chan, C. C. & Detrick, B. Human cytomegalovirus induced cyclooxygenase-2 in human retinal pigment epithelial cells augments viral replication through a prostaglandin pathway. *Microbes Infect* **8**, 2236–2244, doi:S1286-4579(06)00173-010.1016/j.micinf.2006.04.010 (2006). 10.1016/j.micinf.2006.04.010 16782382

[58] Zhu, H., Cong, J. P., Yu, D., Bresnahan, W. A. & Shenk, T. E. Inhibition of cyclooxygenase 2 blocks human cytomegalovirus replication. *Proc Natl Acad Sci USA* **99**, 3932–3937, doi:10.1073/pnas.052713799052713799 [pii] (2002). 10.1073/pnas.052713799 PMC122626 11867761

[59] Melnick, M., Mocarski, E. S., Abichaker, G., Huang, J. & Jaskoll, T. Cytomegalovirus-induced embryopathology: mouse submandibular salivary gland epithelial-mesenchymal ontogeny as a model. *BMC Dev Biol* **6**, 42, doi:1471-213X-6-4210.1186/1471-213X-6-42 (2006). 10.1186/1471-213X-6-42 PMC1601957 16959038

[60] Cheng CY, Huang WR, Chi PI, Chiu HC, Liu HJ (2015). Cell entry of bovine ephemeral fever virus requires activation of Src-JNK-AP1 and PI3K-Akt-NF-kappaB pathways as well as Cox-2-mediated PGE2 /EP receptor signalling to enhance clathrin-mediated virus endocytosis. Cell Microbiol.

[61] Vanarsdall, A. L. & Johnson, D. C. Human cytomegalovirus entry into cells. *Curr Opin Virol* **2**, 37–42, doi:S1879-6257(12)00003-X10.1016/j.coviro.2012.01.001 (2012). 10.1016/j.coviro.2012.01.001 PMC3880194 22440964

[62] Saffert, R. T., Penkert, R. R. & Kalejta, R. F. Cellular and viral control over the initial events of human cytomegalovirus experimental latency in CD34+ cells. *J Virol* **84**, 5594-5604, doi:JVI.00348-1010.1128/JVI.00348-10 (2010). 10.1128/JVI.00348-10 PMC2876595 20335255

[63] Reeves, M. B., Breidenstein, A. & Compton, T. Human cytomegalovirus activation of ERK and myeloid cell leukemia-1 protein correlates with survival of latently infected cells. *Proc Natl Acad Sci USA* **109**, 588–593, doi:111496610810.1073/pnas.1114966108 (2012). 10.1073/pnas.1114966108 PMC3258610 22203987

[64] Leruez-Ville M, Ouachee M, Delarue R, Sauget AS, Blanche S, Buzyn A, Rouzioux C (2003). Monitoring cytomegalovirus infection in adult and pediatric bone marrow transplant recipients by a real-time PCR assay performed with blood plasma. J Clin Microbiol.

[65] Schmittgen TD, Livak KJ (2008). Analyzing real-time PCR data by the comparative C(T) method. Nat Protoc.

[66] Atkinson C, Walter S, Sharland M, Tookey P, Luck S, Peckham C, Griffiths P (2009). Use of stored dried blood spots for retrospective diagnosis of congenital CMV. J Med Virol.

